# Epizootiological investigation of equine herpesvirus type 1 infection among Japanese racehorses before and after the replacement of an inactivated vaccine with a modified live vaccine

**DOI:** 10.1186/s12917-019-2036-0

**Published:** 2019-08-06

**Authors:** Hiroshi Bannai, Koji Tsujimura, Manabu Nemoto, Minoru Ohta, Takashi Yamanaka, Hiroshi Kokado, Tomio Matsumura

**Affiliations:** Equine Research Institute, Japan Racing Association, 1400-4 Shiba, Shimotsuke, Tochigi, 329-0412 Japan

**Keywords:** EHV-1, Inactivated vaccine, Live vaccine, Virus-neutralizing antibody, Vaccination coverage

## Abstract

**Background:**

Equine herpesvirus type 1 (EHV-1) infection is a major cause of pyrexias in winter among Japanese racehorses. In 2014–2015, the Japan Racing Association (JRA) changed the EHV-1 vaccine from an inactivated vaccine to a live vaccine (both produced by Nisseiken). To evaluate the effect of changing the vaccines, the capacities of these vaccines to induce virus-neutralizing (VN) antibodies were compared, and an epizootiological investigation of EHV-1 was performed at the JRA Ritto Training Center during epizootic periods from 2010–2011 to 2016–2017.

**Results:**

Three-year-old horses that received the first dose of live vaccine showed higher geometric mean (GM) VN titers (205 and 220) than those that received inactivated vaccine (83, *P* < 0.05). The response rates after vaccination with the live vaccine (76 and 90%) were higher than that after vaccination with inactivated vaccine (42%, *P* < 0.05). Four-year-old horses from 2015 to 2017 that had received the live vaccine in the previous epizootic periods had higher GM titers (205 to 246) than those from 2011 to 2014, which had received the inactivated vaccine (139 to 164, *P* < 0.05). The estimated numbers of horses infected with EHV-1 or EHV-4, or both, in 2011–2012 (29 [95%CI: 21–37]) and 2013–2014 (37 [95%CI: 27–47]) were higher than those in the other periods (7 [95%CI: 2–12] to 16 [95%CI: 9–23]). Likewise, the seroconversion rates to EHV-1 in horses that stayed at the training center in 2011–2012 (66.0%) and 2013–2014 (52.0%) were higher than those in the other periods (12.0 to 28.6%).

**Conclusions:**

The live EHV-1 vaccine is highly immunogenic and provides greater VN antibody responses than the inactivated vaccine. Unlike the period when the policy was to use inactivated vaccine, there was no detectable epizootic EHV-1 infection at the training center during three consecutive periods after the introduction of the live vaccine. These results suggest that the replacement of inactivated vaccine with live vaccine, together with the achievement of high vaccination coverage, reinforced the herd effect, and contributed to better control of EHV-1 epizootics in the training center.

**Electronic supplementary material:**

The online version of this article (10.1186/s12917-019-2036-0) contains supplementary material, which is available to authorized users.

## Background

Infection with equine herpesviruses types 1 (EHV-1) and 4 (EHV-4) causes respiratory illness in young horses, and EHV-1 infection occasionally causes neurological signs and abortion [1]. Pyrexia caused by EHV-1 epizootics among Japanese racehorses has been an important issue, because it delays training schedules and cause horses to be withdrawn from races [2]. The Japan Racing Association has two training centers (Ritto in western Japan and Miho in eastern Japan), and the spread of EHV-1 in the centers may be attributable to the fact that a number of horses (more than 2000) are kept in close contact and are under stressed conditions from cold weather and hard training. The horses are usually introduced into the training centers at two-year-old, and most of them received first contact with EHV-1 during their first winter there [3]. In contrast, EHV-4 infection is common in breeding and rearing farms [3], hence, most horses in the training centers are already infected with EHV-4 before their arrival. EHV-4 infection in the training centers is generally sporadic and most cases are subclinical [3].

From the epizootic period of 1994–1995, the Japan Racing Association introduced an inactivated vaccine for EHV-1 (Equine Rhinopneumonitis Inactivated Vaccine [Nisseiken]) into the training centers as a way of controlling the epizootic. Because most of the respiratory EHV-1 infection occurred in 3-year-old horses [3], the vaccination policy was targeting this population. However, the policy targeted only about a half of the 3-year-old population due to a budgetary issue, resulting in insufficient control of the epizootic [4]. In 2009–2010, therefore, the vaccination policy was changed, so that all 3-year-old horses could be covered. Our previous study showed that the number of pyretic horses with EHV-1 infection was significantly reduced by achieving high vaccination coverage [4].

In 2014, a modified live EHV-1 vaccine (Equi N Tect ERP [Nisseiken]) was licensed in Japan. The protective efficacy of the live vaccine was reported in challenge studies using a small number of horses [5, 6]. In both studies, vaccinated horses were less likely to have pyrexia and nasal discharge, suggesting that a certain level of protective immunity was acquired by vaccination. In 2014–2015, the vaccination policy for EHV-1 in the training centers changed and the inactivated vaccine was replaced with the live vaccine. We performed an epizootiological investigation of EHV-1 infection at one of our training centers, the Ritto Training Center, to evaluate both the effect of the change in the vaccine and the antibody responses in vaccinated horses.

## Results

### Administrative coverage of EHV-1 vaccine in the 3-year-old population and in the whole population

**Fig. 1:**
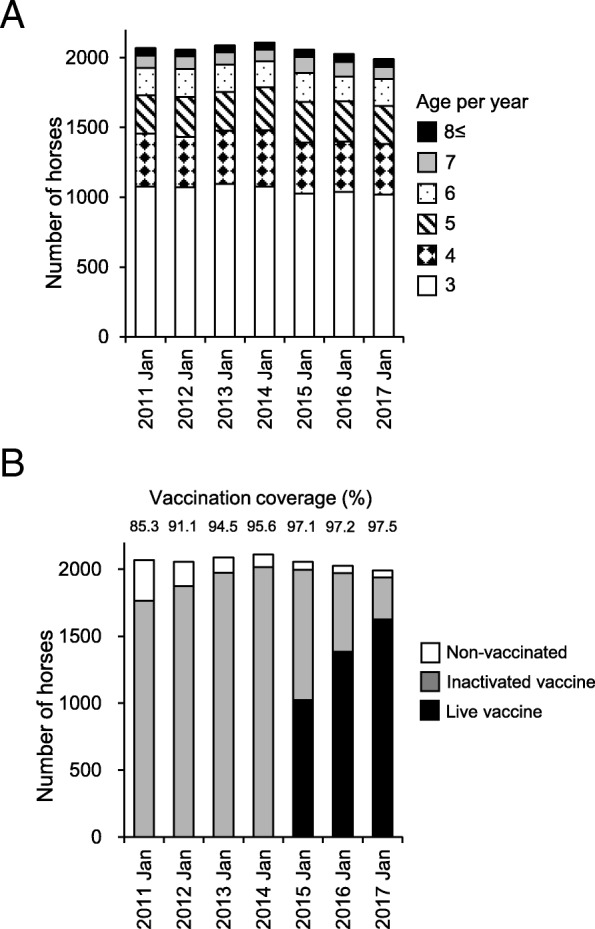
Age distribution of horses in the training center and vaccination coverage with EHV-1 vaccines. **a** Age distribution of horses present in the Ritto Training Center on 1 January in each epizootic period. The numbers of horses classified by age are indicated. **b** Vaccination coverage of EHV-1 vaccines in the whole population at the training center. The numbers of horses vaccinated with inactivated EHV-1 vaccine or live EHV-1 vaccine are indicated. The vaccination coverage of the entire horse population is indicated above the graph

We investigated the coverages of the EHV-1 vaccine in the 3-year-old population and in the whole population of all ages at the training center on 1 January of each epizootic period from 2010–2011 to 2016–2017. The age distributions of the horses in the training center each year are summarized in Fig. 1a. The coverages in the 3-year-old population ranged from 99.1 to 99.8% throughout the investigation periods (data not shown). In the periods where the inactivated vaccine was employed, the coverage of the whole population at the training center increased gradually from 85.3% (2010–2011) to 95.6% (2013–2014) (Fig. 1b). In those periods, all of the vaccinees received inactivated vaccine. After introduction of the live vaccine, the coverage of the whole population continued to be in a high range of 97.1% (2014–2015) to 97.5% (2016–2017) (Fig. 1b). The proportion of the vaccinated horses that received the live vaccine increased gradually as the result of the aging of the population: 51.2% in 2014–2015; 70.3% in 2015–2016; and 83.8% in 2016–2017 (Fig. 1b).

### VN antibody responses of 3-year-old horses inoculated with inactivated or live EHV-1 vaccines

**Fig. 2:**
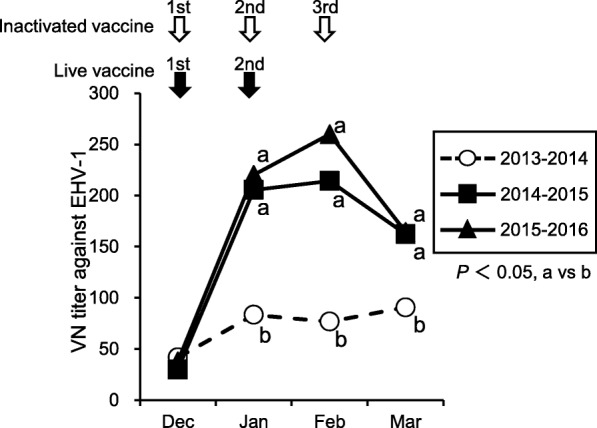
VN antibody titers of 3-year-old horses inoculated with EHV-1 vaccines. Sera were collected from 3-year-old horses (*n* = 50, each period) inoculated with EHV-1 vaccines. In 2013–2014, horses were treated with the inactivated vaccine three times at approximately 1-month intervals. In 2014–2015 and 2015–2016, horses were vaccinated with live vaccine twice with an interval of approximately 1 month. The VN titers were measured for the sequential serum samples, and were expressed as GM titers. a and b, significant differences (*P* < 0.05) between the different symbols

To compare the capacity of inactivated and live EHV-1 vaccines to induce VN antibodies, VN titers were measured for sequential sera from vaccinated horses. In 2013–2014, horses received three doses of inactivated vaccine, a month apart (December, January and February), and in 2014–2015 and 2015–2016, horses received two doses of live vaccine, a month apart (December and January). Horses were confirmed not to be infected with EHV-1 and EHV-4 during the sampling period by using ELISAs for EHV-1 and EHV-4. GM titers at the time of the first vaccination (December) were 42 in 2013, 30 in 2014, and 38 in 2015. In horses that received inactivated vaccine, the GM titer increased to 83 (*P* < 0.05) after the first dose, but no obvious titer rises were observed after the second and third doses (Fig. 2). The numbers of horses for each VN titer in each sampling point were indicated in Additional file 1: Figure S1A. The proportion of individual horses with a ≥ 4-fold titer rise (i.e. the response rate) after the first dose was 22%. The rates after the second and the third doses were 12 and 8%, respectively (Table 1). After stratification by the pre-vaccination titers, 20 out of 30 horses with pre-titers ranging from < 10 to 40 showed significant increases in titers after vaccination with inactivated vaccine, and only one out of 20 horses with pre-titers of ≥80 showed a response (Table 2).

**Table 1:** Response rate in horses (*n* = 50, each period) vaccinated with inactivated or live EHV-1 vaccines

Period	Type of vaccine	Vaccination dose	^a^Response rate after vaccination (%)
2013–2014	Inactivated	1st	22
		2nd	12
		3rd	8
		Total	42
2014–2015	Live	1st	86
		2nd	4
		Total	90^#^
2015–2016	Live	1st	72
		2nd	4
		Total	76^#^

^a^A more than four-fold titer-rise was regarded as a significant increase

^#^Significant difference (*P* < 0.05) with the response rate in 2013–2014

**Table 2:** Relationship between pre-existing VN titer against EHV-1 and its increase after vaccination

Period	Type of vaccine	Response	Response rate (no.) in horses with pre-vaccination VN titer of:								
			< 10	10	20	40	80	160	320	640	Total no.
2013–2014	Inactivated	Significant increase^a^	1	7	7	5	1	0	0	0	21
		No increase	0	1	4	5	9	7	3	0	29
		Total no.	1	8	11	10	10	7	3	0	50
2014–2015	Live	Significant increase	2	12	13	10	5	2	1	0	45
		No increase	0	0	0	0	1	3	1	0	5
		Total no.	2	12	13	10	6	5	2	0	50
2015–2016	Live	Significant increase	7	6	11	6	6	1	1	0	38
		No increase	0	0	0	0	3	3	4	2	12
		Total no.	7	6	11	6	9	4	5	2	50

^a^A more than four-fold titer rise was regarded as a significant increase

The horses that received live vaccine showed much stronger antibody responses than those that received inactivated vaccine, with GM titers of 205 in January 2015 and 220 in January 2016 (*P* < 0.05) (Fig. 2). Although the GM titers after the second dose (214 in February 2015 and 260 in February 2016) were higher than those in January, the differences were not statistically significant, hence an obvious boosting effect after the second dose was not confirmed. GM titers declined greatly from February to March in both periods (162 and 165), although they were still significantly higher than those of horses inoculated with inactivated vaccine (91, *P* < 0.05) (Fig. 2). The numbers of horses for each VN titer in each sampling point were indicated in Additional file 1: Figure S1B and S1C. The response rates after the first dose were 86% in 2014–2015 and 72% in 2015–2016, and those after the second dose were 4% in both periods (Table 1). The total response rates in 2014–2015 and 2015–2016 were 90 and 76%, which were significantly higher than those in 2013–2014 (42%, *P* < 0.05) (Table 1). All horses with pre-titers ranging from < 10 to 40 showed a significant increase in titers after inoculation with live vaccine in both periods (Table 2). Additionally, 8 out of 13 horses in 2014–2015 and 8 out of 20 horses in 2015–2016, which had pre-titers of ≥80, also showed responses after vaccination (Table 2).

### VN antibody titers of 4-year-old horses at the beginning of each epizootic period

**Fig. 3:**
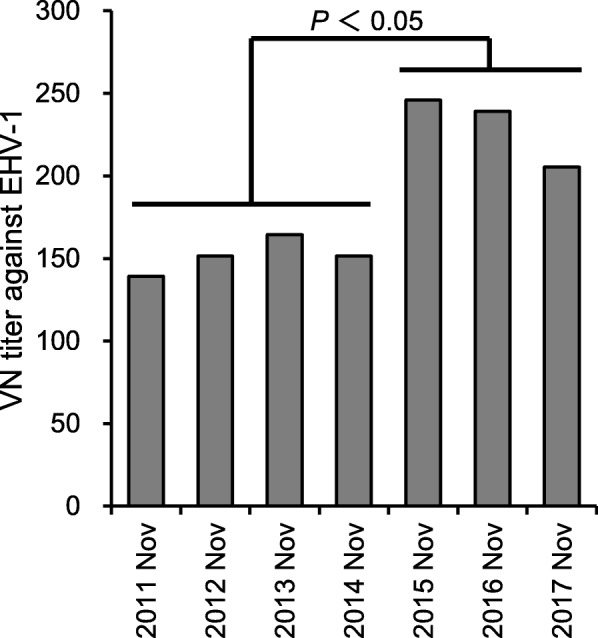
VN antibody titers of 4-year-old horses at the beginning of each epizootic period. VN titers were measured for serum samples collected from 4-year-old horses (*n* = 50, each period) in mid-November in each epizootic period, and were expressed as GM titers. The significant difference (*P* < 0.05) between the grouped periods is indicated

To assess whether the vaccine-induced VN antibodies in the 3-year-old horses remained long-term and influenced their immunity in the next epizootic period, VN titers were measured in the 4-year-old population in mid-November in each period (Fig. 3). The horses had been vaccinated with either inactivated (from 2010–2011 to 2013–2014) or live (from 2014–2015 to 2016–2017) vaccines when they were 3-year-olds in accordance with the vaccination policies. The 4-year-old horses from 2011 to 2014, which corresponded to the population that received the inactivated vaccine, had GM titers that ranged from 139 to 164. The 4-year-old horses from 2015 to 2017, which corresponded to the population that received live vaccine, had GM titers that ranged from 205 to 246, and these were significantly higher than those from 2011 to 2014 (*P* < 0.05). The numbers of horses for each VN titer in each sampling point were indicated in Additional file 2: Figure S2.

### Pyretic horses with EHV-1 or EHV-4 infection, or both, in the training center in winter

**Fig. 4:**
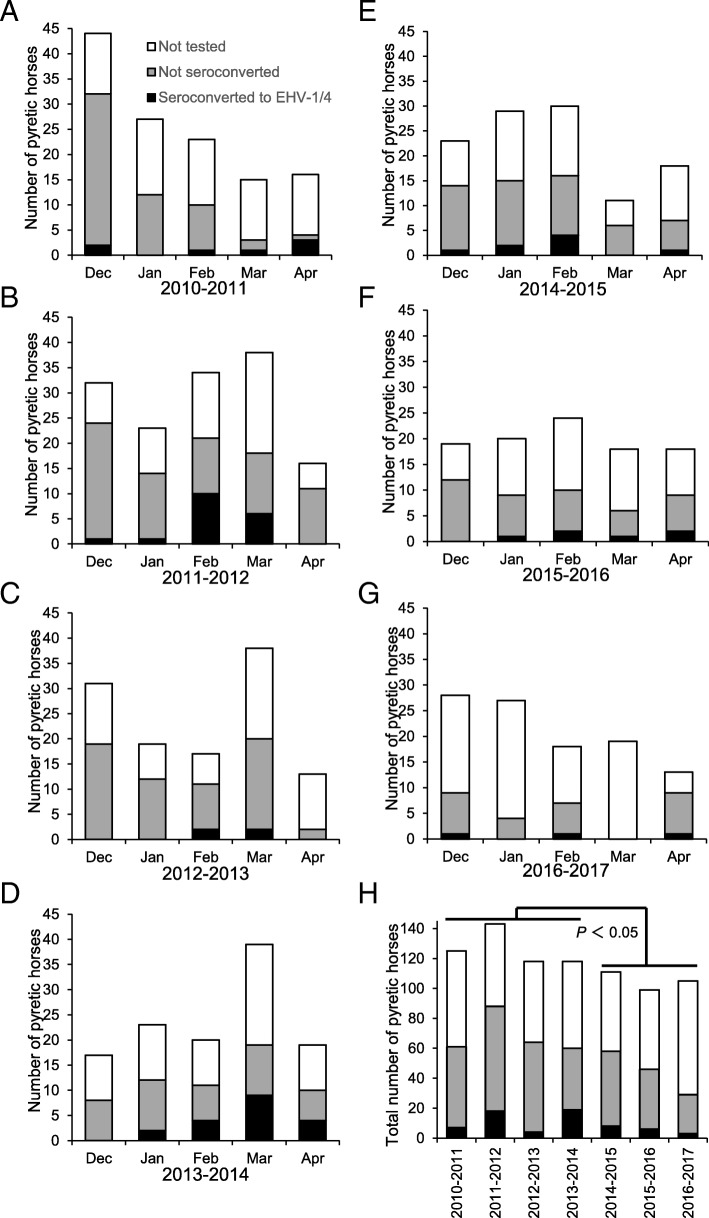
Numbers of pyretic horses that showed seroconversion to EHV-1 or EHV-4, or both in each month of the epizootic periods. **a** 2010–2011. **b** 2011–2012. **c** 2012–2013. **d** 2013–2014. **e** 2014–2015. **f** 2015–2016. **g** 2016–2017. **h** Total numbers in each epizootic period. The significant difference (*P* < 0.05) between the numbers of pyretic horses in the grouped periods is indicated

To assess how the change of vaccine affected the incidence of pyrexia caused by EHV-1 or EHV-4, or both, the pyretic horses were examined with serological tests for EHV-1 and EHV-4 in winter from 2010–2011 to 2016–2017. The numbers of pyretic horses in each month from December to April of each epizootic period are summarized in Fig. 4a-g. When inactivated vaccine was used, pyretic cases were observed with high frequency in some month periods: 44 in December of 2010–2011 (Fig. 4a); 32, 33 and 38 in December; February and March of 2011–2012 (Fig. 4b); 37 in March of 2012–2013 (Fig. 4c); 39 in March of 2013–2014 (Fig. 4d). In contrast, after exchanging the inactivated vaccine with live vaccine in 2014–2015, there were no months with more than 30 pyretic horses (Fig. 4e, f and g). The number of pyretic horses throughout the epizootic period is shown in Fig. 4h. The mean number of pyretic horses in the periods from 2014–2015 to 2016–2017 (105 ± 5) was significantly lower than that in the periods from 2010–2011 to 2013–2014 (126 ± 10, *P* < 0.05).

The results of a CF test and ELISAs specific for EHV-1 and EHV-4 infection revealed that 65 pyretic horses were seroconverted to EHV-1 or EHV-4, or both during the entire investigation period. Among them, 50 cases (76.9%) were EHV-1 infection, 2 cases (3.1%) were EHV-4 infection, and the remaining 13 cases (20.0%) were undiagnosed but included in the numbers of horses infected with EHV-1 or EHV-4, or both. In some of the month periods with more than 30 pyretic horses, the numbers of horses with seroconversion to EHV-1 or EHV-4, or both were higher than those in the other months: 10 and 6 in February and March of 2011–2012 (Fig. 4b); 9 in March of 2013–2014 (Fig. 4d). These results suggested there were epizootic infections with EHV-1 or EHV-4, or both in February and March of 2011–2012 and in March of 2013–2014. In the other month periods, including those after introduction of live vaccine, it appeared that there were no obvious epizootics, and only sporadic cases were observed.

**Fig. 5:**
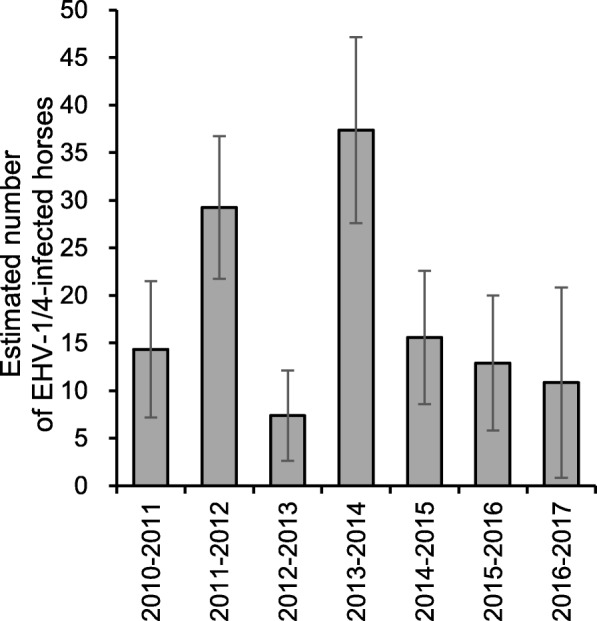
Estimated numbers of horses infected with EHV-1 or EHV-4, or both in each epizootic period. The numbers of infected horses were estimated by multiplying the infection rate with EHV-1 or EHV-4, or both in the paired sera tested by the number of pyretic horses in the corresponding period, and is expressed with error bars of 95% CI

The estimated numbers of horses infected with EHV-1 or EHV-4, or both in each epizootic period are shown in Fig. 5. When inactivated vaccine was used, the estimated numbers of infected horses in 2011–2012 (29 [95% CI: 21–37]) and that in 2013–2014 (37 [95% CI: 27–47]) were higher than those in the other periods (14 [95% CI: 7–21] in 2010–2011 and 7 [95% CI: 2–12] in 2012–2013) (Fig. 5). After the introduction of live vaccine, the estimated numbers of infected horses became stable: 16 (95% CI: 9–23) in 2014–2015, 13 (95% CI: 6–20) in 2015–2016, and 11 (95% CI: 1–21) in 2016–2017 (Fig. 5).

### Infection rate of EHV-1 in the training center in winter

**Fig. 6:**
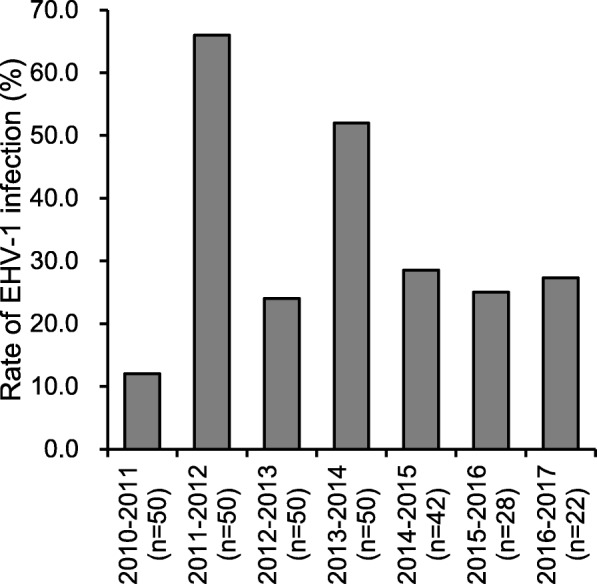
Magnitude of EHV-1 epizootics in the training center in each epizootic period. Paired sera of horses present at the training center throughout the epizootic period (*n* = 50 at maximum) were subjected to EHV-1 gG-ELISA or gE1-peptide-ELISA. The rates of horses that seroconverted to EHV-1 are shown

To assess the magnitude of the EHV-1 epizootics, paired sera from 3-year-old horses that remained at the training center throughout the epizootic period were tested by the ELISAs for EHV-1. When inactivated vaccine was employed, the seroconversion rate in 2011–2012 (66.0%) and that in 2013–2014 (52.0%) were significantly higher than those in the other periods (12.0% in 2010–2011 and 24.0% in 2012–2013, *P* < 0.05) (Fig. 6), suggesting epizootic infections of EHV-1 in these periods. After the introduction of live vaccine, the infection rates were stable: 28.6% in 2014–2015, 25.0% in 2015–2016, and 27.3% in 2016–2017 (Fig. 6). The Pearson’s product moment correlation coefficient for the infection rates among horses that stayed in the training center (Fig. 6) and the estimated numbers of horses infected with EHV-1 or EHV-4, or both (Fig. 5) was 0.824, suggesting these two parameters were highly correlated.

## Discussion

The vaccination policy with the inactivated vaccine targeting all 3-year-old horses, which started in 2009–2010, resulted in coverage of ≥99% of 3-year-old population, and the number of pyretic horses with EHV-1 infection was greatly reduced by the herd effect [4]. One of the important findings in that study was that the herd effect was confirmed only after the second period of the “all 3-year-old” policy (2010–2011) [4]. The mechanism of this delayed effect was considered to be the gradual increase in vaccination coverage in the entire age population, which seemed to satisfy the required value (85.3%) in 2010–2011 [4]. This implies that vaccination coverage not only of the 3-year-old horses but also of the whole population influenced on the herd effect for the control of EHV-1. As shown in the current study, the vaccination coverage in the whole population reached a plateau (≥97%) in 2014–2015 (Fig. 1b). This made the population’s immune status more complete, which constitutes one of the factors for the suppression of EHV-1 epizootics.

The studies by Goehring et al. [7] and Goodman et al. [8] compared the antibody responses in horses after inoculation with inactivated EHV-1 vaccines (Flu-vac Innovator 6 combination vaccine [Fort Dodge], or Pneumabort-K [Pfizer Animal Health]) and a live vaccine (Rhinomune [Pfizer Animal Health]), and they showed that there were no great differences between the VN titers induced with these vaccine products. However, with respect to the two products licensed in Japan (Equi N Tect ERP [Nisseiken] and Equine Rhinopneumonitis Inactivated Vaccine [Nisseiken]), the capacity of the live vaccine to induce VN antibodies was much higher than that of the inactivated vaccine, showing high GM titers and a high response rate even in horses with pre-titers of ≥80 (Fig. 2, Table 1 and Table 2). Thus, the use of a highly immunogenic live vaccine that elicits VN antibodies in the majority of horses will be more effective than inactivated vaccine with which fewer than half the horses showed antibody responses.

According to previous studies of live EHV-1 vaccine [5, 6], the antibody responses after vaccination appeared to be affected by the ages and immune status of the horses. In a study using colostrum-deprived foals [5], the vaccination induced VN antibodies after the first dose, and a booster effect after the second dose was also confirmed. In contrast, when the vaccine was used in 16–21-month-old horses with pre-existing antibodies to EHV-1 and EHV-4 [6], a significant increase was observed in VN titers only after the first dose. Our current result for VN antibody responses in 3-year-old horses was consistent with that observed in the second study [6]: the boosting effect was not obvious (Fig. 2). This was probably because most of the 3-year-old horses in the training center were already infected with EHV-4, which is antigenically cross-reactive with EHV-1 [3], and had a similar immune status to those at 16–21 months of age used in the previous study [6].

Despite the lack of an obvious boosting effect and the declined GM titers observed 2 months after the second vaccination, in March 2015 and 2016 the titers of horses that had received the live vaccine were still higher than those in March 2014 of horses that had received inactivated vaccine (Fig. 2). Also, an investigation of VN titers in the 4-year-old population (Fig. 3) clearly suggested that the difference in the VN titers induced by inactivated and live vaccines in the 3-year-old population was reflected in that of the 4-year-old population in the next epizootic period. Although horses of 4 years old or older, which have probably already been exposed to EHV-1, are considered to be more resistant than young horses, they are also involved in virus circulation [4], and the epizootic infection of EHV-1 in the field usually starts from a re-activation of a latent virus in previously infected horses. In this regard, the immune status of older horses would be important in controlling the epizootic infection of EHV-1, and high VN titers in 4-year-old horses obtained after the introduction of the live vaccine may also be attributed to the suppression of epizootics.

Consistent with a previous report [3], in winter the majority of pyretic horses with seroconversion to the CF test and without a vaccination history during the sampling period were diagnosed as EHV-1 infection. When inactivated vaccine was used from 2010–2011 to 2013–2014, there was an obvious fluctuation in epizootic infections with EHV-1, and the numbers of infected horses increased in February and March of 2011–2012 and March of 2013–2014 (Fig. 4). In contrast, there were no detectable epizootic infections from 2014–2015 onward, and only sporadic cases of infection were observed. Although a significant difference was detected in the numbers of pyretic horses before and after changing the vaccination policies, it is not clear whether the difference could be attributed only to the magnitudes of the EHV-1 epizootics. This was because the seroconversion rates to EHV-1 or EHV-4, or both in some of the month periods with more than 30 pyretic horses under the previous vaccination policy (i.e. December of 2010–2011, December of 2011–2012, and December and March of 2012–2013) were as low as for the other month periods: the cause of this increased number of pyretic horses could not be determined. Nonetheless, epizootic infections with EHV-1 in 2011–2012 and 2013–2014 were also confirmed by the high infection rates among horses that were at the training center during these periods (Fig. 6), regardless of whether the infections were symptomatic or asymptomatic. Because the infection rates among horses throughout the epizootic periods (Fig. 6) showed a high correlation with the estimated numbers of horses infected with EHV-1 or EHV-4, or both (Fig. 5), this parameter accurately reflected the magnitude of EHV-1 epizootics. The continuously low infection rates from 2014–2015 onward was also supportive of the absence of detectable epizootic infection in three consecutive periods after the introduction of live vaccine.

## Conclusions

The live EHV-1 vaccine (Equi N Tect ERP [Nisseiken]) is highly immunogenic and provides greater VN antibody responses than the inactivated vaccine. Unlike the period when the policy was to use inactivated vaccine, there was no detectable epizootic EHV-1 infection at the training center during three consecutive periods after the introduction of the live vaccine. These results suggest that the replacement of inactivated vaccine with live vaccine, together with the achievement of high vaccination coverage, reinforced the herd effect, and contributed to better control of EHV-1 epizootics in the training center.

## Methods

### Horse populations

The study site was the Ritto Training Center of the Japan Racing Association, located in Shiga Prefecture in western Japan, which has a capacity for about 2000 racehorses. Most horses stay at the training center for 1 to 6 months for training and racing. After leaving the center, they are usually reared in other farms for several months for rest and conditioning, and then they re-enter the training center. Approximately 1000 horses are exchanged between the training centers and the farms every month. The age distributions of the horses in the training center on 1 January each year are summarized in Fig. 1a.

### Period of investigation

Epizootic periods are denoted here as 2010–2011, 2011–2012, and so on, to cover the entire period of winter, namely from December to April. In the same way, horses that reached the age of 3 years in January were denoted as the “3-year-old” population, although they were actually 2 years old at the beginning of epizootic periods. The antibody response in the 3-year-old horses after vaccination was investigated for the periods 2013–2014 to 2015–2016. All the other investigations were performed for the periods 2010–2011 to 2016–2017.

### Vaccination policies

From 2010–2011 to 2013–2014, all 3-year-old horses at the training center were inoculated with inactivated EHV-1 vaccine (Equine Rhinopneumonitis Inactivated Vaccine [Nisseiken]) (at least 1 dose, and at most 3 doses, at approximately 1-month intervals). The vaccine contains a formalin-inactivated whole virion of EHV-1 (HH-1 strain, ≥10^8.7^ 50%-tissue-culture infective doses [TCID_50_]/dose) supplemented with aluminum chloride as an adjuvant. The vaccination term during each epizootic period was from mid-December to the end of April.

From 2014–2015 onward, all 3-year-old horses in the training center were inoculated with live EHV-1 vaccine (Equi N Tect ERP [Nisseiken]) (at least 1 dose, and at most 2 doses, at approximately 1-month intervals). The vaccine contained live virus of EHV-1, which lacked the *glycoprotein E* gene (ΔgE-NIBS strain ≥10^4.5^ TCID_50_/dose) without adjuvant. The vaccination term covered more or less the same period as with the previous vaccination policy.

### Administrative coverage of EHV-1 vaccines

The vaccine coverage in the training center was calculated for horses present at the training center on 1 January of each year. The number of horses with a vaccination record (regardless of the number of doses received) in each population was divided by the total number of horses in the corresponding population. The vaccine coverages for the periods 2010–2011 to 2012–2013 are taken from our previous study [4].

### Serological responses of 3-year-old horses inoculated with inactivated or live EHV-1 vaccines

In each epizootic period from 2013–2014 to 2015–2016, 3-year-old horses at the Ritto Training Center were randomly selected. In 2013–2014, they were inoculated with inactivated vaccine three times at 1-month intervals in accordance with the previous vaccination policy. Sera were collected at the time of the first vaccination in December, the second vaccination in January, the third vaccination in February, and 1 month after the third vaccination (March). In 2014–2015 and 2015–2016, the horses were inoculated with live vaccine two times at 1-month intervals in accordance with the current vaccination policy. Sera were collected at the time of the first vaccination in December, the second vaccination in January, and at 1 and 2 months after the second vaccination (February and March). The sera collected in 2013–2014 were subjected to gG-ELISAs for EHV-1 and EHV-4 [9]. The EHV-1 and EHV-4 gG-ELISAs reacts specifically with antibodies induced by EHV-1 and EHV-4 infection, respectively, and not with the antibodies raised after vaccination with the inactivated EHV-1 vaccine [9]. For the sera collected in 2014–2015 and 2015–2016, gE1-peptide-ELISA [10] and gG4-peptide-ELISA [11] were used to detect antibodies to EHV-1 and EHV-4, respectively. The synthetic peptide used in the gE1-peptide-ELISA, which corresponds to a partial amino acid sequence of EHV-1 glycoprotein E, reacts specifically with antibodies induced by EHV-1 infection, and not with antibodies induced by vaccination with live EHV-1 vaccine [10]. The gG4-peptide-ELISA using a synthetic peptide, which corresponds to a partial amino acid sequence of EHV-4 glycoprotein G, was confirmed to have sensitivity and specificity equivalent to those of the EHV-4 gG-ELISA [11]. The horses that were confirmed not to show seroconversion by natural infection with EHV-1 or EHV-4 in either of these tests (*n* = 50, each period) were selected for further analysis. The virus-neutralizing (VN) titer for EHV-1 was measured for the sera by using a focus-reduction method [12]. An antibody response to the vaccination was considered significant if a ≥ 4-fold increase occurred in VN titers between the first sera and any one of the post-vaccination sera.

### VN antibody titers of 4-year-old horses at the beginning of each epizootic period

Four-year-old horses (*n* = 50, each period) in mid-November of each epizootic period from 2011–2012 to 2017–2018 at the Ritto Training Center were randomly selected. They had been vaccinated with inactivated or live EHV-1 vaccines according to the program described above when they were 3 years old in the periods 2010–2011 to 2016–2017, and had stayed at least 90 days at the Ritto Training Center during the period. The VN titers were measured as described above.

### Investigation of pyretic horses with EHV-1 or EHV-4 infection, or both, in the training center in winter

In the epizootic periods from 2010–2011 to 2016–2017 at the Ritto Training Center, the numbers of horses with pyrexia (≥38.5 °C) in each month were monitored, and a high frequency was defined as over 30 pyretic horses detected in a month. For the serological tests, acute sera were taken on the day they developed pyrexia, and after 21- to 35-day intervals, convalescent sera were taken. The convalescent sera could not be collected from some horses, because they had already left the training center at the time of second sampling. The sera collected in the periods 2010–2011 to 2013–2014 were subjected to a complement-fixation (CF) test using EHV-1 HH-1 strain as an antigen [13] and to gG-ELISAs for EHV-1 and EHV-4 [9]. The sera collected in the periods from 2014–2015 onward were subjected to the CF test and to gE1-peptide-ELISA [10] and gG4-peptide-ELISA [11]. Horses that showed a significant increase in titer in the CF test (≥4-fold between the paired sera) and in either EHV-1 gG-ELISA or gE1-peptide-ELISA were diagnosed as infected with EHV-1. Those with a significant increase in titer in the CF test and in either EHV-4 gG-ELISA or gG4-peptide-ELISA were diagnosed as infected with EHV-4. Horses that had a significant increase in titer in the CF test, no increase in either of the ELISAs for EHV-1 and EHV-4, and no history of vaccination during the sampling period were also included in the number of horses infected with EHV-1 or EHV-4, or both. The numbers of horses with pyrexia and those infected with EHV-1 or EHV-4, or both, in the periods 2010–2011 to 2012–2013 are taken from our previous study [4].

For each epizootic period, the number of pyretic horses infected with EHV-1 or EHV-4, or both, was estimated by multiplying the infection rate in tested horses with paired sera by the number of pyretic horses in the period, and was expressed with 95% confidence intervals (CI).

### Investigation of magnitude of EHV-1 epizootics in the training center in winter

Three-year-old horses (*n* = 50 at maximum) were randomly selected from the population that stayed at the training center throughout the winter, and paired sera were collected. Because the number of such horses decreased year by year, the numbers of paired sera collected in the periods from 2014–2015 onward fell to less than 50: *n* = 42 in 2014–2015; *n* = 28 in 2015–2016; *n* = 22 in 2016–2017. First-sera were collected in mid-November and second-sera were collected in mid-May. The sera collected in the periods 2010–2011 to 2013–2014 were tested with EHV-1 gG-ELISA, and those collected in the periods from 2014–2015 onward were tested with gE1-peptide-ELISA. Horses that showed a ≥ 4-fold increase in titer were diagnosed as EHV-1 infected during the epizootic period. The rate of infection with EHV-1 in the periods 2010–2011 to 2012–2013 are taken from our previous study [4].

### Statistical analysis

Two-way analysis of variance and post hoc Tukey’s tests were used to compare the antibody responses after the vaccination of the 3-year-old horses in the different epizootic periods. Wilcoxon’s rank sum test was used to analyze GM titers of 4-year-old horses and the total number of pyretic horses. Fisher’s exact test was used to analyze the response rate after vaccination and infection rate for EHV-1. Pearson’s product moment correlation coefficient value was evaluated according to Guilford’s rule of thumb as follows: < 0.20, slight almost negligible relationship; 0.21 to 0.40, low correlation; 0.41 to 0.70, moderate correlation; 0.71 to 0.90, high correlation; and 0.91≥, very high correlation. The analyses were performed with GraphPad Prism 6 for Windows (GraphPad Software, Inc.). A level of *P* < 0.05 was considered significant.

## Additional files

Additional file 1: **Figure S1.** EHV-1 VN titers of horses vaccinated with the inactivated vaccine (A, 2013–2014) or the modified live vaccine (B, 2014–2015 and C, 2015–2016). Numbers of horses for each VN titers in December, January, February and March were indicated. (PPTX 46 kb)

Additional file 2: **Figure S2.** EHV-1 VN titers of 4-year-old horses. The horses from 2011 to 2014 (A to D) received the inactivated vaccine when they were 3-year-old, and those from 2015 to 2017 (E to G) received the modified live vaccine likewise. Numbers of horses for each VN titers in November were indicated. (PPTX 71 kb)

## Data Availability

The datasets generated and/or analysed during the current study are not publicly available to preserve the individual horses’ and their owners’ privacy, but are available from the corresponding author on reasonable request.

## Abbreviations

CF: Complement-fixation
CI: Confidence intervals
EHV-1: Equine herpesvirus type 1
EHV-4: Equine herpesvirus type 4
GM: Geometric mean
JRA: The Japan Racing Association
TCID_50_: 50%-tissue-culture infective doses
VN: Virus-neutralizing
