# Supplementary material for: Epizootiological investigation of equine herpesvirus type 1 infection among Japanese racehorses before and after the replacement of an inactivated vaccine with a modified live vaccine
Source: BMC Vet Res. 2019 Aug 6;15:280. doi: 10.1186/s12917-019-2036-0 (PMC6683523; doi:10.1186/s12917-019-2036-0)
Supplement: Supplementary file 1 — Figure S1. EHV-1 VN titers of horses vaccinated with the inactivated vaccine (A, 2013–2014) or the modified live vaccine (B, 2014–2015 and C, 2015–2016). Numbers of horses for each VN titers in December, January, February and March were indicated. (PPTX 46 kb) [file 12917_2019_2036_MOESM1_ESM.pptx]

## Slide 1
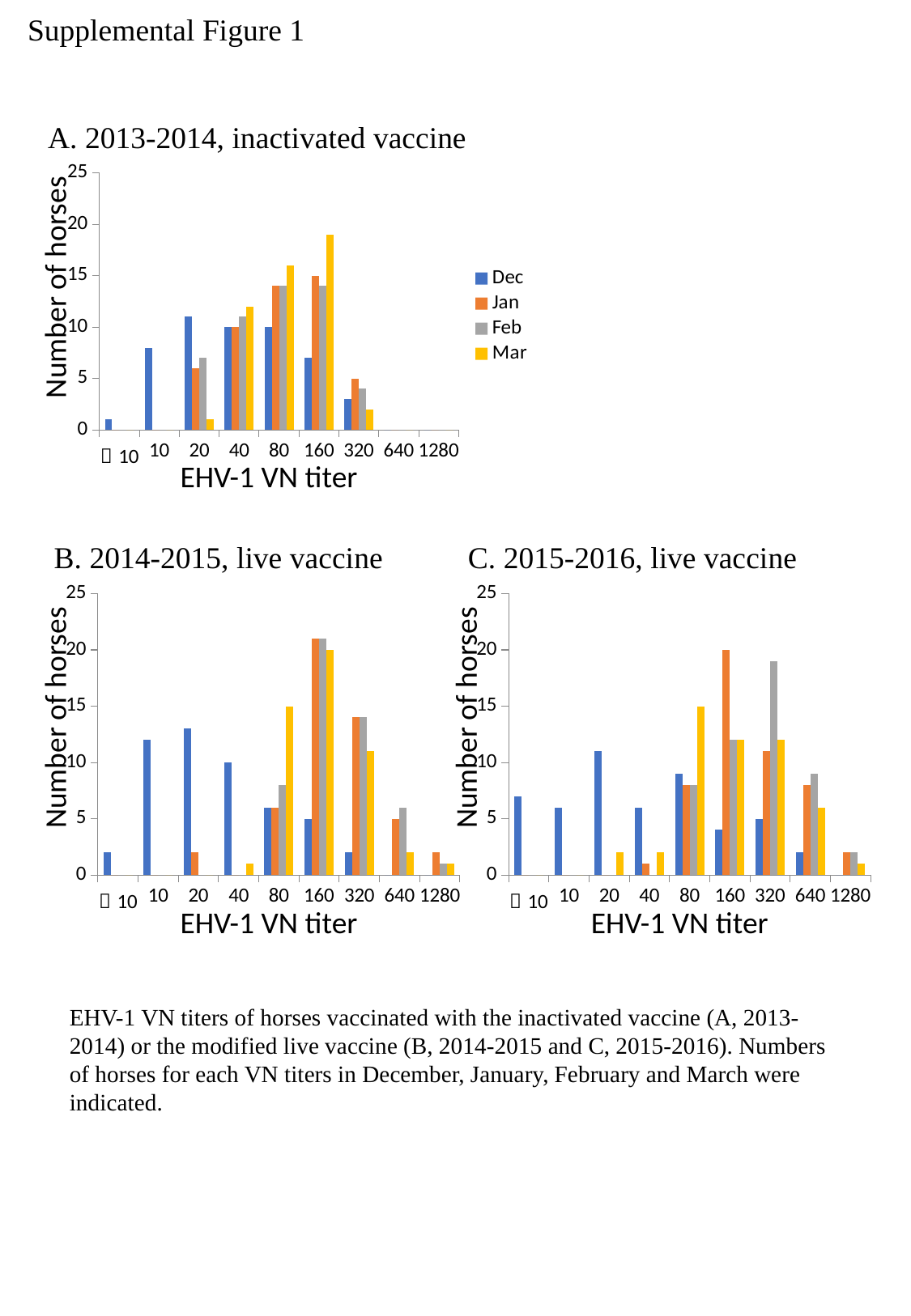

Supplemental Figure 1
A. 2013-2014, inactivated vaccine
### Chart
| Category | Dec | Jan | Feb | Mar |
|---|---|---|---|---|
| ＜10 | 1.0 | 0.0 | 0.0 | 0.0 |
| 10 | 8.0 | 0.0 | 0.0 | 0.0 |
| 20 | 11.0 | 6.0 | 7.0 | 1.0 |
| 40 | 10.0 | 10.0 | 11.0 | 12.0 |
| 80 | 10.0 | 14.0 | 14.0 | 16.0 |
| 160 | 7.0 | 15.0 | 14.0 | 19.0 |
| 320 | 3.0 | 5.0 | 4.0 | 2.0 |
| 640 | 0.0 | 0.0 | 0.0 | 0.0 |
| 1280 | 0.0 | 0.0 | 0.0 | 0.0 |Number of horses
EHV-1 VN titer
B. 2014-2015, live vaccine
C. 2015-2016, live vaccine
### Chart
| Category | Dec | Jan | Feb | Mar |
|---|---|---|---|---|
| ＜10 | 7.0 | 0.0 | 0.0 | 0.0 |
| 10 | 6.0 | 0.0 | 0.0 | 0.0 |
| 20 | 11.0 | 0.0 | 0.0 | 2.0 |
| 40 | 6.0 | 1.0 | 0.0 | 2.0 |
| 80 | 9.0 | 8.0 | 8.0 | 15.0 |
| 160 | 4.0 | 20.0 | 12.0 | 12.0 |
| 320 | 5.0 | 11.0 | 19.0 | 12.0 |
| 640 | 2.0 | 8.0 | 9.0 | 6.0 |
| 1280 | 0.0 | 2.0 | 2.0 | 1.0 |
### Chart
| Category | Dec | Jan | Feb | Mar |
|---|---|---|---|---|
| ＜10 | 2.0 | 0.0 | 0.0 | 0.0 |
| 10 | 12.0 | 0.0 | 0.0 | 0.0 |
| 20 | 13.0 | 2.0 | 0.0 | 0.0 |
| 40 | 10.0 | 0.0 | 0.0 | 1.0 |
| 80 | 6.0 | 6.0 | 8.0 | 15.0 |
| 160 | 5.0 | 21.0 | 21.0 | 20.0 |
| 320 | 2.0 | 14.0 | 14.0 | 11.0 |
| 640 | 0.0 | 5.0 | 6.0 | 2.0 |
| 1280 | 0.0 | 2.0 | 1.0 | 1.0 |Number of horses
Number of horses
EHV-1 VN titer
EHV-1 VN titer
EHV-1 VN titers of horses vaccinated with the inactivated vaccine (A, 2013-2014) or the modified live vaccine (B, 2014-2015 and C, 2015-2016). Numbers of horses for each VN titers in December, January, February and March were indicated.
