# Supplementary material for: Epizootiological investigation of equine herpesvirus type 1 infection among Japanese racehorses before and after the replacement of an inactivated vaccine with a modified live vaccine
Source: BMC Vet Res. 2019 Aug 6;15:280. doi: 10.1186/s12917-019-2036-0 (PMC6683523; doi:10.1186/s12917-019-2036-0)
Supplement: Supplementary file 2 — Figure S2. EHV-1 VN titers of 4-year-old horses. The horses from 2011 to 2014 (A to D) received the inactivated vaccine when they were 3-year-old, and those from 2015 to 2017 (E to G) received the modified live vaccine likewise. Numbers of horses for each VN titers in November were indicated. (PPTX 71 kb) [file 12917_2019_2036_MOESM2_ESM.pptx]

## Slide 1
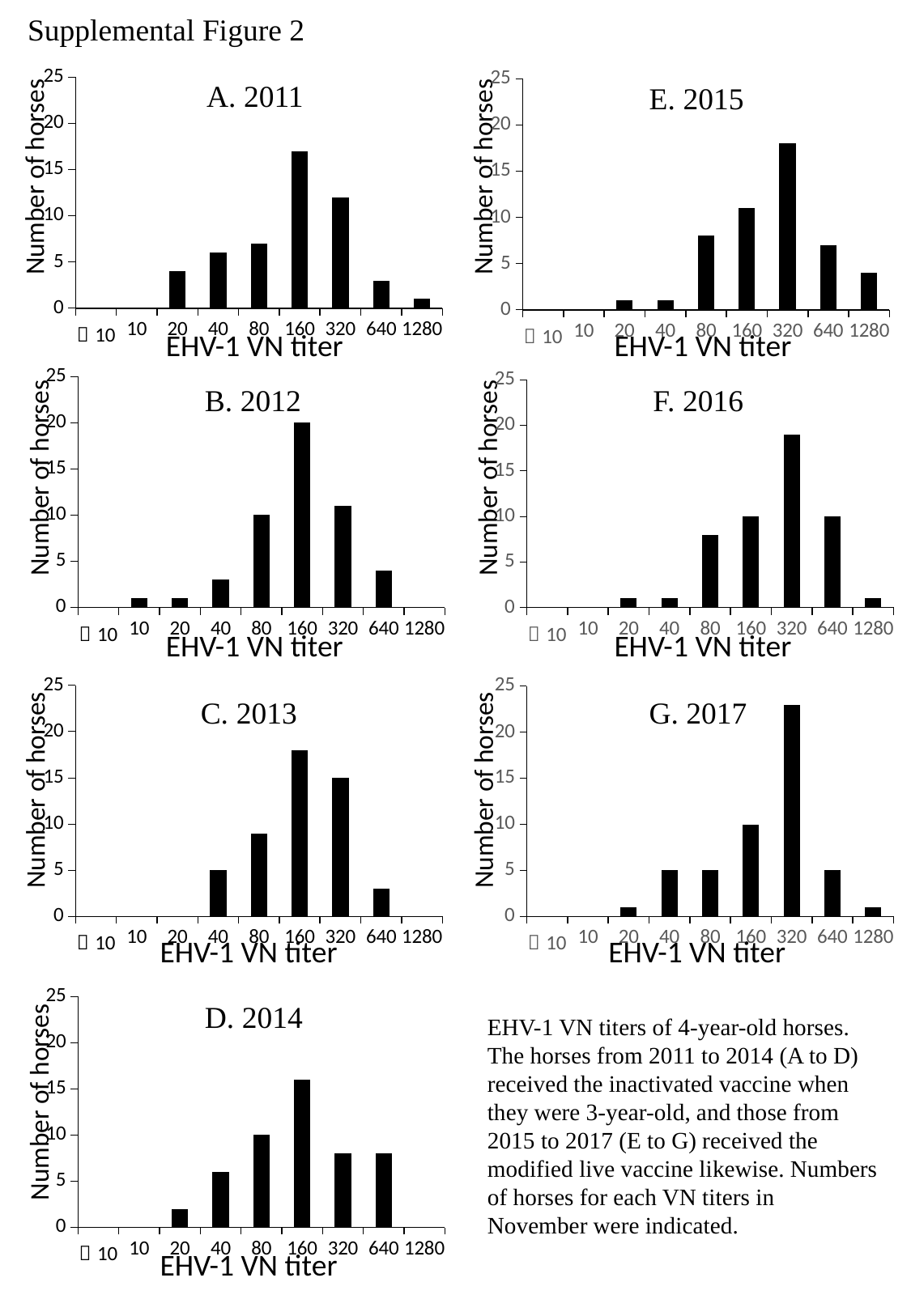

Supplemental Figure 2
### Chart
| Category | |
|---|---|
| ＜10 | 0.0 |
| 10 | 0.0 |
| 20 | 4.0 |
| 40 | 6.0 |
| 80 | 7.0 |
| 160 | 17.0 |
| 320 | 12.0 |
| 640 | 3.0 |
| 1280 | 1.0 |
### Chart
| Category | |
|---|---|
| ＜10 | 0.0 |
| 10 | 0.0 |
| 20 | 1.0 |
| 40 | 1.0 |
| 80 | 8.0 |
| 160 | 11.0 |
| 320 | 18.0 |
| 640 | 7.0 |
| 1280 | 4.0 |A. 2011
E. 2015
Number of horses
Number of horses
EHV-1 VN titer
EHV-1 VN titer
### Chart
| Category | |
|---|---|
| ＜10 | 0.0 |
| 10 | 1.0 |
| 20 | 1.0 |
| 40 | 3.0 |
| 80 | 10.0 |
| 160 | 20.0 |
| 320 | 11.0 |
| 640 | 4.0 |
| 1280 | 0.0 |
### Chart
| Category | |
|---|---|
| ＜10 | 0.0 |
| 10 | 0.0 |
| 20 | 1.0 |
| 40 | 1.0 |
| 80 | 8.0 |
| 160 | 10.0 |
| 320 | 19.0 |
| 640 | 10.0 |
| 1280 | 1.0 |F. 2016
B. 2012
Number of horses
Number of horses
EHV-1 VN titer
EHV-1 VN titer
### Chart
| Category | |
|---|---|
| ＜10 | 0.0 |
| 10 | 0.0 |
| 20 | 0.0 |
| 40 | 5.0 |
| 80 | 9.0 |
| 160 | 18.0 |
| 320 | 15.0 |
| 640 | 3.0 |
| 1280 | 0.0 |
### Chart
| Category | |
|---|---|
| ＜10 | 0.0 |
| 10 | 0.0 |
| 20 | 1.0 |
| 40 | 5.0 |
| 80 | 5.0 |
| 160 | 10.0 |
| 320 | 23.0 |
| 640 | 5.0 |
| 1280 | 1.0 |G. 2017
C. 2013
Number of horses
Number of horses
EHV-1 VN titer
EHV-1 VN titer
### Chart
| Category | |
|---|---|
| ＜10 | 0.0 |
| 10 | 0.0 |
| 20 | 2.0 |
| 40 | 6.0 |
| 80 | 10.0 |
| 160 | 16.0 |
| 320 | 8.0 |
| 640 | 8.0 |
| 1280 | 0.0 |D. 2014
EHV-1 VN titers of 4-year-old horses. The horses from 2011 to 2014 (A to D) received the inactivated vaccine when they were 3-year-old, and those from 2015 to 2017 (E to G) received the modified live vaccine likewise. Numbers of horses for each VN titers in November were indicated.
Number of horses
EHV-1 VN titer
